# Use of family planning methods in Kassala, Eastern Sudan

**DOI:** 10.1186/1756-0500-4-43

**Published:** 2011-02-28

**Authors:** Abdel Aziem A Ali, Duria A Rayis, Mona Mamoun, Ishag Adam

**Affiliations:** 1 Faculty of Medicine, Kassala University, Sudan; 2 Faculty of Medicine, University of Khartoum, Sudan

## Abstract

**Background:**

Investigating use and determinants of family planning methods may be instructive in the design of interventions to improve reproductive health services.

**Findings:**

Across sectional community-based study was conducted during the period February-April 2010 to investigate the use of family planning in Kassala, eastern Sudan. Structured questionnaires were used to gather socio-demographic data and use of family planning. The mean ± SD of the age and parity of 613 enrolled women was 31.1 ± 7 years and 3.4 ± 1.9, respectively. Only 44.0% of these women had previously or currently used one or more of the family planning methods. Combined pills (46.7%) and progesterone injection (17.8%) were the predominant method used by the investigated women. While age, residence were not associated with the use of family planning, parity (> five), couple education (≥ secondary level) were significantly associated with the use of family planning. Husband objection and religious beliefs were the main reasons of non-use of family planning.

**Conclusion:**

Education, encouragement of health education programs and involvement of the religious persons might promote family planning in eastern Sudan.

## Findings

## Introduction

High fertility rate and inadequate spacing between births, can lead to high maternal and infant mortality. An estimated 600 000 maternal deaths occur worldwide each year; the vast majority of these take place in developing countries. WHO estimates that 13% of these deaths are due to unsafe abortion. Worldwide, where approximately 50 million women resort to induced abortion, frequently results in high maternal morbidity and mortality [1,2]. Thus, family planning and spacing among births are one of the methods to avoid these deaths. Promotion of family planning and contraceptive use is highly adopted by the international community as one of the strategy to reduce the maternal mortality and to reach the Millennium Development Goals [3-5]. Africa characterized by high rate of lack to contraceptive access reaching 57% and this lack lead to unwanted pregnancies, increased demand to abortion and death related to unsafe abortion [6].

In Sudan, the largest African country, there is unaccepted high maternal mortality [7,8]. Moreover, legally, politically and culturally access to abortion create internal dispute, therefore effective contraceptive programming should be the current and future approach to reduce the risk and unwanted pregnancies. Few published data exist concerning use of family planning services in Sudan [9] especially eastern part where we have recently observed high maternal morbidity and mortality in this setting [10]. Thus, the current study was conducted to investigate use of family planning methods among married women in Kassala, Eastern Sudan.

## Materials and methods

A community-based cross sectional household survey was conducted to investigate use and factors associated with family planning services in Kassala, Eastern Sudan between February and April 2010. Kassala is 550 killometer from Khartoum on Ethiopian-Eritrean border with 500.000 inhabitants. In Kassala there are 28 health centers and three hospitals providing health services and there is an office of Sudanese family planning association providing different aspect of services like pills and intrauterine contraceptive device free of charge. After taking an informed consent, pre-tested structured questionnaires in the local Arabic language were administered by a trained medical officer to gather data of women in the reproductive age (12-49 years) in privacy. Those women who have been pregnant before were inquired to identify family planning experiences and associated factors (age, parity, education and husband education). Utilization was defined as the respondents' state of using or having used one or more family planning methods (oral combined contraceptive pills, intrauterine contraceptive device, injections, male condoms, female condoms, and female sterilization). Traditional methods inquired for were breastfeeding (lactational amenorrhea) and rhythm.

### Statistics

Sample size was calculated with 3 percentage points of the true proportion, assuming the true proportion was 70% and that 10% of women would not be respond. Data were entered into computer database and double checked before analysis. The proportion of women who used family planning was calculated. Univariate and multivariate analyses were conducted using family planning as dependent factor and age, parity, residence and the education level of women and their husbands as independent variables. Confidence interval of 95% was calculated and *P <*0.05 was considered significant.

### Ethics

The study received ethical clearance from the Health Research Board Committee at Kassala State Ministry of Health, eastern Sudan.

## Results

The mean (SD) age and parity of 613 investigated women (their parity ranges between 1-10) was 31.1 ± 7 years and 3.4 ± 1.9, respectively. Around one third (30.8%) of these women had ≥ secondary education level which equal eight years. Only 44.0% of these women had previously or currently used one or more of the family planning methods. Oral combined contraceptive pills was the predominant method used by the enrolled women (46.7%) followed by progesterone injection (17.8%), progesterone only pills (15.2%), intrauterine contraceptive device (7.4%), safe period (6.7%), male condom (4%) and abstinence (2.2%), table 1.

**Table 1:** Methods of contraception used by women in Kassala, eastern Sudan*

Method of contraception	Numbers of women	Percentage
Oral combined pills	126	46.7%
Progesterone injection	048	17.8%
Progesterone only pills	041	15.2%
Intrauterine device	020	07.4%
Safe period	018	06.7%
Male condom	011	04%
abstinence	006	02.2%
Total	270	100%

*Data were shown as n (%).

Husband objection (47.5%), religious belief (28.2%), desire for more babies (14.3%), fear of side effects (6.14%), non-availability (2.7%) and medical diseases (1.2%) were common reason for non use of family planning given by the respondents.

While age and residence were not associated with the use of family planning services, high parity (> five), educational levels (≥ secondary level) of the couples were the significant predictors of family planning services use in this setting, table 2.

**Table 2:** Factors associated with use of family planning in Kassala, Sudan using univariate and multivariate analyses

Variable	Univariate analyses			Multivariate analyses		
	**OR**	**95% CI**	***P*-value**	**OR**	**95% CI**	***P*-value**
Age, years	1.0	0.9-1.0	0.7	0.9	0.9-1.0	0.6
Parity > 5	1.9	1.2-3.0	0.005	3.2	1.5-6.7	0.002
Women's education ≥ secondary level	2.4	8.5-22.0	< 0.001	8.8	5.0-15.2	< 0.001
Husband's education≥ secondary level	9.5	5.8-15.5	< 0.001	5.1	2.9-9.1	< 0.001
Rural residence	1.9	0.9-2.3	0.06	1.2	0.7-1.2	0.4

Abbreviations: OR, Odds Ratio; CI, confidence interval

## Discussion

The main findings of the current study were; poor use of family planning services, combined oral pills, progesterone injection and pills were the most common used methods and couples education were significantly associated with use of family planning in this setting. Although, in Sudan there are various culture and traditions that differ with the locations, the main results of this study were in line with our recent observations in Darfur, Western Sudan [9]. In Darfur, the use of family planning services was 34.2%, contraceptive pills was the most frequently used method (74.4%), and the common reasons given by respondents for not using these services were, wanted more children, and fearing side effects [9]. Many researches have been carried-out to determine the influential factors that motivate women to adopt contraceptive method [11]. In neighboring Tanzania, it has been reported that, men have strong influence over fertility decision; therefore some women are using contraception secretly [11]. Thus, when considering the influential determinants for family planning services in our setting, husband approval should be considered by the health care providers. Women in developing countries should be encourage to put their own decision concerning the reproductive health services and this was obvious in the current study since husband objections (47.5%) represented the main reason given by the respondents for not using contraception. Furthermore, the impact of beliefs in personal and community health practice is very strong and might not be scientifically true. However, involvement of the religious persons might increase the utilization of family planning since these beliefs accounted for 28.2% in our study.

We have shown that oral contraceptive pills was the most common method of family planning in western Sudan as well as in the current study [9]. In neighboring Ethiopia Injectable form were the most commonly preferred modern contraceptive (63.2%) followed by oral contraceptive pills (21.2%). Few women (9.5%) reported a preference for the use of condoms [12]. This point (few women preferred condom) is of paramount importance to consider in preventing HIV and sexually transmitted diseases in the setting. The Ethiopian expertise documented that family planning can be integrated into HIV voluntary counseling and testing clinics [13]. In Uganda it has been shown that, expanding family planning services can substantially contribute towards Mother to Child Transmission [14]. However, policy-makers and programme managers should carefully consider the characteristics and reproductive health needs of target populations when making decisions about service integration. Yet, it might be difficult to integrate and expand family planning services into HIV like other African countries because Sudanese women had low incidence of HIV and had poor uptake for HIV testing and counseling [15,16].

Couple's education in many African countries as well as in the current study was associated with utilization of family planning [17]. Unfortunately there is high level of illiteracy among Sudanese women and illiteracy was the significant predictor for maternal morbidity and mortality in the different regions of Sudan including the eastern part [18,19].

## Conclusion

In summary the study showed low use of family planning. Couples education was the main predictor of this use. Husband non approval and religious belief were influential determinants for family planning. Thus improving health education and participation of religious persons might promote family planning in this setting.

## Competing interests

The authors declare that they have no competing interests.

## Authors' contributions

AAA, DAR, MM and MB carried out the study and participated in the statistical analysis and procedures. ID and DAR coordinated and participated in the design of the study, statistical analysis and the drafting of the manuscript. All the authors read and approved the final version.
